# Antibiofilm Effects of Novel Compounds in Otitis Media Treatment: Systematic Review

**DOI:** 10.3390/ijms252312841

**Published:** 2024-11-29

**Authors:** Ana Jotic, Katarina Savic Vujovic, Andja Cirkovic, Dragana D. Božić, Snezana Brkic, Nikola Subotic, Bojana Bukurov, Aleksa Korugic, Ivana Cirkovic

**Affiliations:** 1 Clinic for Otorhinolaryngology and Maxillofacial Surgery, University Clinical Center Serbia, Pasterova 2, 11000 Belgrade, Serbia; ana.jotic@med.bg.ac.rs (A.J.); bojana.bukurov@med.bg.ac.rs (B.B.); korugicaleksa@gmail.com (A.K.); 2 Faculty of Medicine, University of Belgrade, Dr Subotica 1, 11000 Belgrade, Serbia; nikola.subotic@med.bg.ac.rs; 3 Department of Pharmacology, Clinical Pharmacology and Toxicology, Faculty of Medicine, University of Belgrade, Dr Subotica 1, 11000 Belgrade, Serbia; katarina.savic-vujovic@med.bg.ac.rs; 4 Institute for Medical Statistics and Informatics, Faculty of Medicine, University of Belgrade, 11000 Belgrade, Serbia; andja.cirkovic@med.bg.ac.rs; 5 Department of Microbiology and Immunology, Faculty of Pharmacy, University of Belgrade, Vojvode Stepe 450, 11000 Belgrade, Serbia; dragana.bozic@pharmacy.bg.ac.rs; 6 Institute for Laboratory Diagnostics “Konzilijum”, Sv. Save 28a, 11000 Belgrade, Serbia; brkic.snezana@gmail.com; 7 Institute of Microbiology and Immunology, Faculty of Medicine, University of Belgrade, Dr Subotica 1, 11000 Belgrade, Serbia

**Keywords:** otitis media, novel compounds, antibiofilm effect, clinical bacterial isolates, bacterial biofilm

## Abstract

Otitis media (OM) is a frequent disease with incidence rate of 5300 cases per 100,000 people. Recent studies showed that polymicrobial biofilm formation represents a significant pathogenic mechanism in recurrent and chronic forms of OM. Biofilm enables bacteria to resist antibiotics that would typically be recommended in guidelines, contributing to the ineffectiveness of current antimicrobial strategies. Given the challenges of successfully treating bacterial biofilms, there is an growing interest in identifying novel and effective compounds to overcome antibacterial resistance. The objective of this review was to provide an overview of the novel compounds with antibiofilm effects on bacterial biofilm formed by clinical isolates of OM. The systematic review included studies that evaluated antibiofilm effect of novel natural or synthetic compounds on bacterial biofilm formed from clinical isolates obtained from patients with OM. The eligibility criteria were defined using the PICOS system: (P) Population: all human patients with bacterial OM; (I) Intervention: novel natural or synthetic compound with biofilm effect; (C) Control standard therapeutic antimicrobial agents or untreated biofilms, (O) Outcome: antibiofilm effect (biofilm inhibition, biofilm eradication), (S) Study design. The PRISMA protocol for systematic reviews and meta-analysis was followed. From 3564 potentially eligible studies, 1817 duplicates were removed, and 1705 were excluded according to defined exclusion criteria. A total of 41 studies with available full texts were retrieved by two independent authors. Fifteen articles were selected for inclusion in the systematic review which included 125 patients with OM. A total of 17 different novel compounds were examined, including N-acetyl-L-cysteine (NAC), tea tree oil, xylitol, eugenol, Aloe barbadensis, Zingiber officinale, Curcuma longa, Acacia arabica, antisense peptide nucleic acids, probiotics *Streptococcus salivarius* and *Streptococcus oralis*, Sodium 2-mercaptoethanesulfonate (MESNA), bioactive glass, green synthesized copper oxide nanoparticles, radish, silver nanoparticles and acetic acid. *Staphylococcus aureus* was the most commonly studied pathogen, followed by *Pseudomonas aeruginosa* and *Haemophilus influenzae*. Biofilm inhibition only by an examined compound was assessed in six studies; biofilm eradication in four studies, and both biofilm inhibition and biofilm eradication were examined in five studies. This systematic review indicates that some compounds like NAC, prebiotics, nanoparticles and MESNA that have significant effects on biofilm are safe and could be researched more extensively for further clinical use. However, a lack of data about reliable and efficient compounds used in therapy of different types of otitis media still remains in the literature.

## 1. Introduction

Otitis media (OM) is defined as an infection of the middle ear, which includes acute and chronic forms of the disease. In 2019, the recorded incidence of OM was 5300 in 100,000 people [1]. The global burden of the disease is significant. OM may present a significant cause of preventable hearing loss, especially in developing countries, with long-term effects on communication, educational and work progress, and patients’ quality of life. Consequences of the OM include suppurative intratemporal or intracranial complications (facial nerve paralysis, labyrinthitis, mastoiditis, meningitis, epidural, subdural, and brain abscess formation) that could be life-threatening [2,3]. These pathogens share certain systemic complications like bacteremia, sepsis, and immune-mediated complications, especially in at-risk populations (like infants, the elderly, and immunocompromised individuals) [4,5]. Also, OM is the most common indication for antimicrobial prescription, with a reported antibiotic prescribing rate of 85.6% in the pediatric population [6,7]. This is already resulting in the development of multi-drug resistance of most common otopathogens (*Streptococcus pneumoniae* and *Haemophilus influenzae*) due to penicillin non-susceptibility and ampicillin-resistant strains, respectively [8].

The pathophysiology of OM is a result of a complex interaction between predominant microbial pathogens, local inflammation, the host’s immune response, genetic predisposition, and environmental factors. Recent studies showed that polymicrobial biofilm formation by most common otopathogens (*H. influenzae*, *S. pneumoniae*, *Staphylococcus aureus*, *Moraxella catarrhalis*) represents a significant pathogenic mechanism in recurrent and chronic forms of otitis media [9]. Biofilm formation enables the bacteria to express up to a 1000-fold increase in antibiotic resistance compared to their planktonic forms [10]. The existing resistance of biofilms to antimicrobial agents is attributed to the protective extracellular matrix material surrounding the bacteria, their reduced metabolic activity, and the distinct biofilm phenotype adopted through quorum sensing or lateral gene transfer [11]. These factors enable bacteria to resist antibiotics that would typically be recommended in guidelines, contributing to the ineffectiveness of current antimicrobial strategies [12].

Despite significant advances in understanding the pathophysiology of OM, several gaps remain in the literature regarding the antibiofilm effects of potential treatments. Although biofilms are known for their high resistance to antibiotics and immune clearance, the precise molecular mechanisms driving this resistance in OM are still not well understood. Most studies on antibiofilm agents rely heavily on in vitro models, which fail to fully replicate the complex environment of the middle ear. Furthermore, there is a notable lack of robust in vivo models capable of accurately mimicking biofilm formation and persistence in OM, making it challenging to translate research findings into clinical application [13]. OM is often caused by polymicrobial infections, yet many studies still focus on single-species biofilms. The interactions between different microbial species within biofilms and their collective resistance to treatment can influence the efficacy of antibiofilm compounds in polymicrobial infections and limit the development of broad-spectrum antibiofilm strategies [14]. Also, host factors (like microbiome, inflammation, immune response, etc.) influence biofilm formation, persistence, and treatment of OM [15,16].

Given these challenges associated with the successful treatment of bacterial biofilms, there is a growing interest in exploring novel and effective compounds to overcome antibacterial resistance. New research focuses on the investigation of natural products, synthetic compounds, and combination therapies that can either inhibit biofilm formation or eradicate preformed biofilm. These advancements could improve clinical outcomes, and address the growing concern of biofilm-associated infections in OM treatment. Our hypothesis is that novel compounds exhibit significant antibiofilm activity against bacterial biofilms formed by clinical isolates from OM, potentially outperforming or enhancing the activity of traditional antimicrobial treatments in disrupting biofilm structure and reducing biofilm-associated bacterial viability.

This review aims to provide an overview of the novel compounds with antibiofilm effects on bacterial biofilm formed by clinical isolates from patients with OM.

## 2. Materials and Methods

### 2.1. Study Design

The PRISMA protocol for systematic reviews and meta-analysis was followed in order to perform our research [17]. This review was registered at the PROSPERO international register of systematic reviews with the number 554979.

### 2.2. Eligibility Criteria

The systematic review included studies that evaluated the antibiofilm effect of novel natural or synthetic compounds on bacterial biofilm, formed from clinical isolates taken from patients with OM. The eligibility criteria were defined according to previously composed PICOS system: (P) Population: all human patients with bacterial OM (OM was defined as acute of chronic mucosal inflammation of the middle ear cleft, mastoid and Eustachian tube), (I) Intervention: novel natural or synthetic compound with antibiofilm effect (N-acetylcysteine, bacteriophages, peptides, probiotics, nanoparticles, nanozymes, ceragenins, exopolysaccharides, *Bacillus licheniformis*, plant extracts, novel quorum sensing inhibitor yd 47, Dornase alfa, garlic extract, medical device coatings, d-methionine, eugenol, 5-azacytidine, manuka honey, nitric oxide, pyrimidinedione, bioengineered honey, 2-mercaptoethane sulfonate (MESNA), amniotic membrane extract, chorionic membrane extract, Sinefungin, Ethylenediaminetetraacetic acid (EDTA), riboswitch, etc.), (C) Control: standard therapeutic antimicrobial agents or untreated biofilms, (O) Outcome: antibiofilm effect (biofilm inhibition, biofilm eradication), (S) Study design: experimental study, controlled trials, prospective or retrospective cohort design, nested case-control in cohort design, case-control design, and cross-sectional design. Reasons for exclusion were defined as follows: (1) Foreign language—if the article was not published in English; (2) Wrong publication type—if the publication was not an original article (narrative review, systematic review, meta-analysis, editorial, comment, letter to the editor, guideline, case report, case series, abstract, conference poster without full-text article, etc.); (2) Wrong population was stated—in studies when the evaluated cases were not from the human population with otitis media; (3) Wrong method—the situation when there was no biofilm detection; (4) Wrong outcome—defined as no antibiofilm effect evaluated, and (5) Wrong topic—stated in case the the article did not suit any of inclusion criteria. Two researchers with expertise in conducting systematic reviews and meta-analyses (A.C., I.C.) developed and ran the search in three electronic databases: PubMed, Web of Science (WoS), and SCOPUS until 19 March 2024. The search queries used in PubMed Web of Science and SCOPUS are presented in detail in Table 1. In addition, reference lists of articles identified through electronic retrieval and relevant reviews and editorials were manually searched to check for more potentially relevant articles.

### 2.3. Article Screening and Selection

Publications were screened for inclusion in the systematic review independently by two reviewers (A.J., K.S.V.) first through title and abstract and afterword through full-text reading. All disagreements were resolved by discussion at each stage with the inclusion of a third reviewer (I.C.). Cohen’s Kappa coefficient was calculated between authors involved in blinded article selection within the title and abstract step, a value of 0.42 was deemed to reflect moderate agreement between the authors. Studies were included in the full-text screening step if either reviewer identified the study as potentially eligible or if the abstract and title did not have sufficient information for exclusion.

### 2.4. Data Abstraction and Quality Assessment

Two researchers (A.J., K.S.V.) independently abstracted the following (1) study characteristics: author(s), year of publication, country of research, study design, type of research (in vivo or in vitro), (2) novel compound characteristics, (3) cases characteristics (number of cases, age, gender, OM type (acute, chronic or recurrent), the causative bacteria, therapy except antibiofilm agent, (4) objective and method of objective evaluation, and (5) main findings: biofilm inhibition and/or biofilm eradication. Independent reviewers used previously designed protocols in selecting and abstracting data. Authors of relevant articles were contacted to obtain unavailable manuscripts and/or missing data. The quality of the included studies was evaluated by an adapted version of the Newcastle–Ottawa tool (NOS) for observational studies [18]. The study quality, according to NOS, was good (3 or 4 stars in selection AND 1 or 2 stars in comparability AND 2 or 3 stars in outcome/exposure domain, or ≥7 stars in total), fair (2 stars in selection AND 1 or 2 stars in comparability AND 2 or 3 stars in outcome/exposure domain, or 5–6 stars in total), or poor (0 or 1 star in selection OR 0 stars in comparability OR 0 or 1 star in outcome/exposure, or ≤4 stars in total). Jadad could have 5 points in total, and the categorization was as follows: 0–2: low and 3–5: high quality. The risk of bias was assessed by the Joanna Briggs Institute tool using the checklist for quasi-experimental studies [19].

### 2.5. Statistical Analysis

Numerical data were presented as the arithmetic mean with standard deviation or as median with range, depending on the data distribution. Normality was assessed by the Shapiro—Wilk test. Categorical data were reported as absolute with relative number in percentages.

## 3. Results

### 3.1. Study Selection

**Figure 1:**
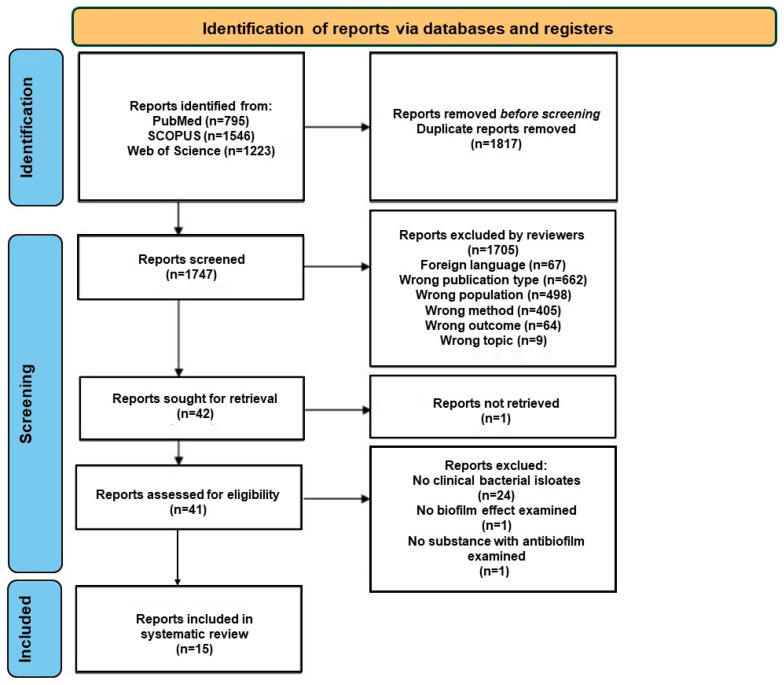
PRISMA flow diagram depicting the process followed for the selection of the studies.

A total of 3564 potentially eligible articles were found. Titles and abstracts were evaluated for 1747 articles after duplicates (1817) were removed. According to previously defined exclusion criteria, a total of 1705 were excluded (Supplementary Material: Table S1. Excluded studies), while 41 were retrieved with available full texts. Finally, 15 articles were selected for inclusion in the systematic review. A flow diagram illustrating the selection process is presented in Figure 1.

### 3.2. Study Characteristics

Characteristics of 15 included publications within the systematic review are presented in Table 2. They were published between 2007 and 2023, with a known 125 patients with OM, although five studies did not report the number of respondents suffering from OM. The minimum sample size was one, and the maximum was 29. Almost all studies were experimental in vitro with clinical samples (14/15), while just one had a case-control design and in vivo type of research. Three studies were conducted in South Korea, two in Iraq, and one study each was conducted in Finland, Pakistan, Spain, the United States of America, Italy, Mexico, Norway, Egypt, Serbia, and Indonesia. The origin of the examined population was heterogeneous. Five studies originated from Europe, four from Asia, two from America, and one from Africa. The age of respondents was reported in two out of all included studies. Children were the target population in 2/15. Gender was reported in only one study with the male: female ratio of 4:1. A total of 17 different novel compounds were examined in 15 included studies. N-acetyl-L-cystein (NAC) was evaluated in three studies, alone in two and in combination with dry propolis extracts in one. Other examined novel compounds were as follows: tea tree oil, xylitol, eugenol alone and eugenol with carvacrol, aloe barbadensis (aloe vera), Zingiber officinale (ginger), Curcuma longa (turmeric), Acacia arabica (kikar) plant crude extracts, antisense peptide nucleic acids (PNAs), probiotics *Streptococcus salivarius* and *Streptococcus oralis*, Sodium 2-mercaptoethanesulfonate (MESNA), bioactive glass (BAG), green synthesized copper oxide nanoparticles (CuO NPs), radish, silver nanoparticles (AgNPs), and acetic acid. The most commonly evaluated type of OM was chronic otitis in six studies, of which two examined a chronic suppurative form of otitis. Recurrent and acute otitis were examined in one study each. The type of otitis was not specified in the seven included publications. The antibiofilm effect (biofilm inhibition and/or biofilm eradication) of novel compounds was evaluated on 28 different causative agents that were isolated from clinical samples. *S. aureus* was most often isolated (in eight out of 15 studies), then *P. aeruginosa* (in seven out of 15 studies), and *H. influenzae* (in four out of 15 studies). Six studies included in the review examined the antibiofilm effect on one bacterium, while the other nine examined the antibiofilm effect on multiple bacteria. Biofilm inhibition only by an examined compound was assessed in six studies; biofilm eradication only was examined in four studies, while both biofilm inhibition and biofilm eradication were examined in five studies. It was established that the examined compound had a dose-dependent effect in seven studies. All studies were experimental in vitro studies, except one case-control in vivo study, and were assessed as studies of low quality.

### 3.3. Risk of Bias Within Studies

Obtained results regarding the level of risk of bias within the included studies are presented in Table 3. The JBI risk of bias tool recommends the interpretation of the level of risk of bias within studies as follows: < 49% corresponds to high, 50–69% to moderate, and > 70% to low risk of bias. There were no included studies with a high level of risk of bias, while there were ten with moderate and five with a low level of risk of bias.

### 3.4. Feasibility Assessment of Meta-Analysis

A detailed feasibility assessment of the meta-analysis is presented in Table 4.

## 4. Discussion

The estimated global OM incidence rate was 10.85%, or 709 million cases per year, where 51% of cases occurred in children younger than five years [2]. OM represents a progressive continuum of infectious and inflammatory conditions affecting the spaces of the middle ear. It often starts as an acute OM (AOM) which is defined as acute inflammation in the middle ear with rapid onset of otalgia and fever. Episodes of otitis could be repeated over time. Recurrent acute OM (rAOM) is defined as three or more well-documented and separate AOM episodes for six months, or four or more episodes for 12 months, with at least one episode in the past six months [35]. As a result of repeated episodes of acute otitis followed by recurrent infections of other parts of the upper respiratory tract (nose and the nasopharynx), fluid can persist in the middle ear without signs or symptoms of acute ear infection, which is defined as OM with effusion (OME) [36]. In some cases, these earlier forms of the disease may progress to chronic otitis media (COM), defined as a chronic inflammation of the middle ear mucosa with tympanic membrane perforation, with or without persistent otorrhea, lasting at least six weeks or more.

The study that first detected the presence of otopathogens (such as *S. pneumoniae*) in biofilms on the middle ear mucosa in children with COM was published two decades ago [37]. Since then, both polymicrobial bacterial biofilms and single-species intracellular biofilms were detected on the middle ear mucosa and within the middle ear effusion of patients with rAOM, OME and COM [38]. Biofilms are now recognized as a significant factor contributing to the persistence of inflammation and infection in OM [39]. Clinically, biofilm’s resistance mechanisms pose a challenge in treating the disease, especially when they are associated with tissues that cannot be physically removed [11]. Currently, there are no effective treatments or preventative therapies against biofilm. As a result, efforts are increasingly focused on developing new compounds aimed at inhibiting biofilm formation and/or eradicating existing biofilms.

### 4.1. Plant-Derived Extracts

Surprisingly, only a small number of studies examining the effect of novel compounds on biofilm met the criteria to be included in this systematic review. The research mostly includes plant derived extracts like tea tree oil, eugenol (clove oil), carvacrol (oregano constituent), Aloe barbadensis (aloe vera), Zingiber officinale (ginger), Curcuma longa (turmeric), and Acacia arabica (kikar) and radish (Raphanus sativus) [20,22,23,31]. Tea-tree oil demonstrated an ability to inhibit the biofilm formation of MRSA isolates on the surface of tympanostomy tubes in vitro [20]. Yadav et al. established that 0.01%, 0.02%, 0.04%, and 0.08% eugenol inhibited MRSA and MSSA biofilm formation and eradicated preformed biofilm in vitro and in vivo OM rat models [22]. Rehman et al. examined the effects of extracts from four plants (Aloe barbadensis, Zingiber officinale, Curcuma longa, Acacia arabica) on *P. aeruginosa*, *Staphylococcus haemolyticus* and *Staphylococcus hominis* biofilm in vitro [23]. A. arabica was most effective in the inhibition of bacterial biofilm formation, particularly on *P. aeruginosa* biofilm. Raphanus sativus inhibited the biofilm formation of *S. aureus*, *Citrobacter* spp., *Proteus* spp., *Klebsiella* spp., *Pseudomonas* spp., *Enterobacter* spp., *E. coli*, *Morganella* spp., and *Aeromonas* spp. obtained from ear swabs of chronic otitis [31]. In all these studies, the mechanism of action was not described. The biofilm effect of these plant extracts was investigated in other studies involving resistant clinical and ATCC isolates from other sites (vancomycin-resistant enterococci, methicillin-resistant *S. aureus* (MRSA), broad-spectrum β-lactamase-producing *E. coli* and *P. aeruginosa* [40,41,42,43,44,45].

### 4.2. N-Acetyl Cysteine

NAC was already identified as an antibiofilm agent. In vitro studies demonstrated its role in different stages of biofilm formation, like adhesion to surfaces, matrix production and organization, and dispersal of preformed biofilms of Gram-negative and Gram-positive bacteria and yeasts [46]. This systematic review included three in vitro studies where samples were taken from patients with acute and chronic suppurative OM. Domenech et al. demonstrated the eradication of mixed biofilm formed by non-encapsulated *S. pneumoniae*, non-typeable (NT) *H. influenzae* collected from patients with AOM [24]. Resistant bacterial biofilm formed by, e.g., MRSA and quinolone-resistant *P. aeruginosa* (QRPA), was susceptible to NAC, and the effect was dose-dependent. NAC significantly decreased bacterial adhesion, inhibited biofilm formation, and increased biofilm eradication in samples collected in patients with refractory post-tympanostomy tube otorrhea [28]. Bozic et al. demonstrated biofilm formation inhibition and biofilm eradication formed by all relevant otopathogens *S. aureus*, *M. catarrhalis*, *S. pneumoniae*, *S. epidermidis*, *P. aeruginosa*, and *H. influenzae*, where samples were taken from COM mucosa of surgically treated patients [33]. NAC can easily be combined with other substances like propolis or antibiotics, with an enhanced synergy effect, making it even more effective as a biofilm treatment agent [47,48].

### 4.3. Sodium 2-Mercaptoethanesulfonate

Sodium 2-mercaptoethanesulfonate (MESNA) is an interesting compound that is already used in the surgery of patients with COM with cholesteatoma and atelectatic forms of COM. These clinical studies indicate that MESNA can help in the intraoperative elimination of the disease and reduce rates of recurrent disease in patients with cholesteatoma [49,50]. Its effect on biofilm involves the inhibition of the early stages of biofilm adhesion and formation, disruption of mature biofilm membranes, reduction of extracellular protein, and suppression of the expression of genes related to adhesion proteins and exopolysaccharide production [27]. These findings are particularly relevant to OM with cholesteatoma, considering the important role bacterial biofilms play in the disease’s resistance to antimicrobial agents, recurrence, and aggressiveness.

### 4.4. Peptide Nucleic Acids

Peptide nucleic acids (PNAs) are synthetic molecules similar to DNA/RNA that can mediate gene silencing by antigen or antisense action [51]. This manifests with the ability to inhibit the transcription of a target gene by binding to complementary DNA sequence, or as the ability to inhibit the translation of a target gene by binding to the mRNA. PNA-peptides exhibit antimicrobial activity by inhibiting the expression of genes essential for bacterial viability, thus demonstrating antibiofilm effects [52]. Otsuka et al. demonstrated non-typeable *H. influenzae* biofilm eradication by PNA in middle ear fluid and nasopharyngeal isolates collected from patients with chronic otitis media [25]. Minimum biofilm eradication concentrations (MBEC) varied from 45 mg/L (10 µmol/L) to 179 mg/L (40 µmol/L) in different clinical isolates. This indicates that no significant conclusions about the behavior of isolates in the biofilm formation process and the antibiofilm effect of used compounds can be made based on one clinical isolate. PNAs have shown promise in species-specific inhibition of targeted pathogens without disrupting non-targeted bacteria or affecting normal microflora, making them good antimicrobial agent candidates [53].

### 4.5. Nanoparticles

Health-related applications of nanotechnology are emerging, and the synthesis of antimicrobial nanoparticles seems a promising strategy for developing novel biofilm-controlling agents. Antibiofilm activity and antimicrobial activities of nanoparticles composed of metals (silver, zinc, iron, copper, titanium, gold, and magnesium) against multidrug-resistant *Klebsiella* spp., *Pseudomonas* spp., *E. coli*, and MRSA were demonstrated in previous studies [54]. The use of nanostructures as carriers for antimicrobial agents improves bioavailability reduces the likelihood of sub-therapeutic drug accumulation and minimizes drug-related toxicity [55]. Copper oxide nanoparticles had an inhibitory effect on biofilm formed by *S. aureus*, *P. aeruginosa*, *K. pneumoniae*, *S. epidermidis*, *E. coli*, *P. vulgaris*, *C. freundii*, *E. cloacae*, *H. influenzae*, *P. oryzihabitans* isolates from patients with COM [30]. Silver nanoparticles also had an inhibitory effect on biofilm formed by bacterial isolates (*B. cereus*, *P. aeruginosa*) [32]. The probable mechanism of action involves physical disruption of biofilm, interference with bacterial adhesion, and quorum sensing [55]. In addition, this study demonstrated antifungal activity of silver nanoparticles on *P. chrysogenum*, *A niger*, and *A. flavus*. The authors concluded that alterations in the microbial flora of OM and biofilm formation contributed to reduced antibiotic efficacy and the emergence of multidrug-resistant pathogens.

### 4.6. Probiotics

Probiotics *S. salivarius* 24 SMB and *S. oralis* 89a inhibited biofilm formation and eradicated biofilm formed by bacterial isolates (*S. aureus*, *S. epidermidis*, *S. pyogenes*, *S. pneumoniae*, *M. catarrhalis*, *P. acnes*) collected from patients with upper respiratory tract infections, including OM [26]. With the increasing difficulty of discovering new effective means of treatment, the local application of probiotics found their use in the prevention of recurrent infections of the upper respiratory tract, including acute and recurrent otitis media [56,57]. The antimicrobial activity of *S. salivarius* 24SMBc is based on its ability to adhere to epithelial cells. Also, blpU bacteriocin-production by *S. salivarius* 24SMB, mediates intra- and interspecies competition with inhibitory activity against *S. pyogenes* and *S. pneumoniae* and provides a competitive advantage in colonization in vivo [58].

### 4.7. Bioactive Glass

Bioactive glass S53P4 (BAG) has been used in reconstructive otosurgery for years [59]. Its antimicrobial and antibiofilm properties have been studied. Strong biofilm reduction produced by a broad range of microorganisms, such as *S. aureus*, *P. aeruginosa*, *K. pneumoniae*, *A. baumannii* and *S. epidermidis* was established by multiple studies [60]. However, there was only one study that demonstrated the ability of BAG-S53P4 to eradicate biofilm formed by *S. aureus* isolated from patients with chronic suppurative OM [29]. The antibacterial properties of BAG are based on its granules’ interaction with body fluids, exchanging ions and raising the local pH, thus creating an alkaline environment. This, along with the release of sodium, silica, calcium, and phosphate ions, increased salt concentration and osmotic pressure and effectively inhibited bacterial growth and prevented bacterial adhesion [61].

### 4.8. Acetic Acid

Acetic acid had a *P. aeruginosa* antibiofilm effect formed by bacterial isolates collected from five patients with chronic suppurative OM [34]. In this study, the compound was tested in vitro, while there are few previous studies that prove the ototoxicity of acetic acid in guinea and chinchilla animal models, making it unusable in clinical settings [62,63].

### 4.9. Toxic Effect of the Compounds Included in the Systematic Review

Ototoxicity is an important characteristic of a compound considered as an antibiofilm agent in OM. There are limited data about this trait for most of the compounds included in the systematic review. NAC was proved not to be ototoxic and even had a curative effect on hearing loss. These effects of NAC indicate that it could be a very promising agent in treating recurrent and resistant middle ear infections with systemic and local administration [64,65]. MESNA did not induce any changes in audiological results in 55 patients who underwent otologic surgery with topical administration of MESNA into the middle ear [66]. On the other hand, tee tree oil ototoxicity was examined in animal models, with opposite results. While ototoxicity was confirmed in the high-frequency region of the cochlea in a guinea pig model, the effect was not proven in a chinchilla model [67,68]. There are no data in the literature on ototoxicity after local application of the other plant extracts included in this review (Aloe barbadensis, Zingiber officinale, Curcuma longa, and Acacia arabica), but there are reports of positive action on hearing loss when using them as oral supplements [69,70].

Acetic acid has an ototoxic effect, and it is not recommended for use in patients with a perforated eardrum [71]. Nanoparticles are viewed as excellent carriers for anti-inflammatory drugs, pro-degradation mediators, and even gene therapy. Because of these characteristics, they have already found use in the treatment of drug- and noise-induced hearing loss and tinnitus [72,73]. Ototoxicity can be an issue in silver and copper nanoparticles since one study noted toxic effects of both AgNPs and CuNPs on hair cells of zebrafish embryos that were dose-dependent [74]. Bioactive glass is a reconstructive material that was extensively used in otosurgery and is not oto- and vestibulotoxic. This was proved in animal models and numerous clinical studies [75,76,77].

Some of the review compounds could have systemic toxic effects. There are growing concerns about the toxicity of nanoparticles since inflammatory, cytotoxic, genotoxic, and neurotoxic effects have been demonstrated. Nanoparticles already have wide biomedical applications, so future research and design of nanoparticles is aimed at overcoming this effect [78,79]. Plant-based compounds like aloe vera were linked with toxicity, genotoxicity, and carcinogenicity in some forms [80]. Cytotoxicity, genotoxicity, and immunotoxicity of eugenol was demonstrated in animal models and in vitro studies, but it is generally considered safe for human use [81]. Curcuma longa (turmeric) and tea tree oil can have gastrointestinal adverse effects on the liver when consumed in higher doses [82,83].

### 4.10. Limitations of the Study

One of the possible limitations could be the inclusion of research only performed on clinical OM isolates, not on laboratory and reference strains. There are positive aspects of using ATCC bacterial strains, like standardization and consistent properties, reproducibility in experiments, and traceability with detailed genetic, variety, and phenotypic information, history, and growth conditions. On the other hand, bacteria from ATCC collections may not evolve under specific laboratory conditions as wild-type strains, potentially limiting their relevance in biofilm studies. ATCC strains are often maintained in optimal laboratory conditions, which may not accurately reflect the complex behavior of bacteria in natural or clinical environments. Additionally, this could also be accompanied by genetic drift affecting their original characteristics [84,85]. As a result, these strains could have different biofilm-forming capacities in vivo and in vitro, and therefore, results should be interpreted with care.

Studies included in the review according to the determined criteria were all in vitro studies, except for one in vivo study. There are only a limited number of studies in which a meaningful number of biofilm model systems were systematically compared, although these models do not specifically refer to OM [13]. The expression of certain virulence genes can vary significantly depending on the biofilm model used. This means that the choice of model can impact the results, and findings from one model system may not necessarily apply to another [86].

The review could not address the duration of effectiveness of these compounds in preventing biofilm recurrence. Also, no conclusion can be made about the effect of the reviewed compounds on a broader spectrum of otopathogens since most of them tested only one or a small number of isolates.

### 4.11. Recommendations for Future Research

As we collected all articles evaluating the antibiofilm effect of novel natural or synthetic compounds on bacterial biofilm, formed from clinical isolates obtained from patients with OM with heterogeneous types of compounds, study population, causative agents, defined outcomes and method of their evaluation, as well as different level of risk of bias, without adequate control groups, this systematic review encourages researchers to perform future studies in this field.

Current treatment modalities often fail to effectively address the chronicity of OM. Novel compounds reviewed in this paper could reduce recurrence, minimize antibiotic resistance due to repeated antibiotic use, decrease the need for surgical treatment, and improve the quality of life.

It is necessary to standardize the doses or concentrations of new substances for antibiofilm testing in further research. Only 5/15 of the papers included in this systematic review [22,24,25,33,34] determined minimum inhibitory concentration (MIC), minimum biofilm inhibitory concentration (MBIC) and minimum biofilm eradication concentration (MBEC) of the compounds. This would allow us to evaluate antimicrobial efficacy, optimize the dosage, and standardize results across future research.

Further research should focus on combining approaches, which involve the simultaneous use of compounds with different mechanisms of antibiofilm effect to degrade extracellular polymeric substances, disperse biofilm, and eliminate persister cells [87]. This would potentially lead to long-term effects of used antibiofilm compounds. Another possible approach to enhance outcomes would be the integration of antibiofilm compounds with conventional therapies (such as antibiotics and surgical interventions) [87,88].

Finally, an obvious direction for future research is clinical trials evaluating the effect of antibiofilm compounds on OM patients. Such trials would address the limitations of in vitro studies, like controlled conditions and the absence of a complex multifactorial environment of human OM, including co-morbidities, diverse microbiome, and treatment compliance. Clinical trials would also overcome limitations of in vivo studies, like variations between animal models and possible inaccurate pathophysiology and biofilm ecology.

## 5. Conclusions

This systematic review highlights the lack of data on reliable and effective compounds for the treatment of different types of OM. The studies included in the review are mostly experimental in vitro studies (with the exception of one case-control study) and were conducted on a small number of human OM isolates. Data about the patients included in these studies, such as age, gender, and comorbidities, were mostly missing. Therefore, any conclusions or recommendations regarding biofilm treatment in patients based on these data would be premature. However, compounds like NAC, prebiotics, nanoparticles, and MESNA could be researched more extensively for further clinical use.

## Figures and Tables

**Table 1:** Search queries used in the systematic review.

Electronic Database	Search Strategy
PubMed	(OM or middle ear inflammation) and (biofilm* or bacteria* biofilm* or antibiofilm or anti-biofilm or antibiofilm effect* or anti-biofilm effect* or natural compound* or natural product* or synthetic compound* or synthetic product* or N-acetylcysteine or NAC or Bacteriophage* or Peptide* Probiotic* or Nanoparticle* or Nanozyme* or Exopolysaccharides or *Bacillus licheniformis* or Plant Extract* or novel quorum sensing inhibitor yd 47 or Dornase alfa or garlic extract or d-methionine or eugenol or 5-azacytidine or manuka honey or nitric oxide or pyrimidinedione or bioengineered honey or MESNA or 2-mercaptoethane sulfonate or Amniotic membrane extract or Chorionic membrane extract or Sinefungin or EDTA or Ethylenediaminetetraacetic acid or Riboswitch or Photodynamic therapy)
Web of Science	TS = “otitis media” AND (TS = “biofilm*” OR TS = “bacteria* biofilm*” OR TS = “antibiofilm” OR TS = “anti-biofilm” OR TS = “antibiofilm effect*” OR TS = “anti-biofilm effect*” OR TS = “natural compound*” OR TS = “natural product*” OR TS = “synthetic compound*” OR TS = “synthetic product*” OR TS = “N-acetylcysteine” OR TS = “NAC” OR TS = “Bacteriophage*” OR TS = “Peptide*” OR TS = “Probiotic*” OR TS = “Nanoparticle*” OR TS = “Nanozyme*” OR TS = “Exopolysaccharides” OR TS = “Bacillus licheniformis” OR TS = “Plant Extract*” OR TS = “Novel quorum sensing inhibitor yd 47” OR TS = “Dornase alfa” OR TS = “garlic extract” OR TS = “d-methionine” OR TS = “eugenol” or TS = “5-azacytidine” OR TS = “manuka honey” OR TS = “nitric oxide” OR TS = “pyrimidinedione” OR TS = “bioengineered honey” OR TS = “MESNA” OR TS = “2-mercaptoethane sulfonate” OR TS = “Amniotic membrane extract” OR TS = “Chorionic membrane extract” OR TS = “Sinefungin” OR TS = “EDTA” OR TS = “Ethylenediaminetetraacetic acid” OR TS = “Riboswitch” OR TS = “Photodynamic therapy”)
SCOPUS	(TITLE-ABS-KEY (“otitis media”) OR TITLE-ABS-KEY (“Middle ear inflammation”)) AND (TITLE-ABS-KEY (“Biofilm*”) OR TITLE-ABS-KEY (“Bacteria* biofilm*”) OR TITLE-ABS-KEY (“antibiofilm”) OR TITLE-ABS-KEY (“anti-biofilm”) OR TITLE-ABS-KEY (“antibiofilm effect*”) OR TITLE-ABS-KEY (“anti-biofilm effect*”) OR TITLE-ABS-KEY (“natural compound*”) OR TITLE-ABS-KEY (“natural product*”) OR TITLE-ABS-KEY (“synthetic compound*”) OR TITLE-ABS-KEY (“synthetic product*”) OR TITLE-ABS-KEY (“N-acetylcysteine”) OR TITLE-ABS-KEY (“NAC”) OR TITLE-ABS-KEY (“Bacteriophage*”) OR TITLE-ABS-KEY (“Peptide*”) OR TITLE-ABS-KEY (“Probiotic*”) OR TITLE-ABS-KEY (“Nanoparticle*”) OR (“Nanozyme*”) OR TITLE-ABS-KEY (“Exopolysaccharide*”) OR TITLE-ABS-KEY (“Bacillus licheniformis”) OR TITLE-ABS-KEY (“Plant extract*”) OR TITLE-ABS-KEY (“novel quorum sensing inhibitor yd 47”) OR TITLE-ABS-KEY (“Dornase alfa”) OR TITLE-ABS-KEY (“garlic extract*”) OR TITLE-ABS-KEY (“d-methionine”) OR TITLE-ABS-KEY (“Eugenol”) OR TITLE-ABS-KEY (“5-azacytidine”) OR TITLE-ABS-KEY (“manuka honey”) OR TITLE-ABS-KEY (“nitric oxide”) OR TITLE-ABS-KEY (“Pyrimidione”) OR TITLE-ABS-KEY (“Bioengineered honey”) OR TITLE-ABS-KEY (“MESNA”) OR TITLE-ABS-KEY (“2-mercaptoethane sulfonate”) OR TITLE-ABS-KEY (“Amniotic membrane extract*”) OR TITLE-ABS-KEY (“Chorionic membrane extract*”) OR TITLE-ABS-KEY (“Sinefugin”) OR TITLE-ABS-KEY (“EDTA”) OR TITLE-ABS-KEY (“Ethylenediaminetetraacetic acid”) OR TITLE-ABS-KEY (“Riboswitch”) OR TITLE-ABS-KEY (“Photodynamic therapy”))

*, wildcard symbol used to include variations of the root word.

**Table 2:** Systematic review study characteristics.

Author, Year Country	Study Design Type of Research	Study Quality	Substance (Characteristics)	*n* Age Gender	Type of Otitis Media Sample Cause	Objective Method of Objective Evaluation	Main Findings
Park, 2007 South Korea [20]	Experimental study In vitro	Low	Tea-tree oil (Essential oil from leaves of the indigenous Australian plant, *Melaleuca alternifolia* or tea tree)	n = 6 100% tea-tree oil (n = 3) 50% tea-tree (n = 3) NR NR	Chronic suppurative with otorrhea Ear fluid for culture was collected from the external auditory canal using a swab MRSA	Biofilm formation inhibition on the surface of the tympanostomy tubes SEM	1. 100% tea tree oil—considerable reduction in the density and biofilm structures 2. 50% diluted tea tree oil—destruction of the surface and partial reduction of the biofilm
Kurola, 2010 Finland [21]	Experimental study (part of the randomized clinical trial for the surgical prevention of recurrent otitis media) In vitro	Low	0.5% xylitol (Five-carbon sugar alcohol)	n = 20 NR NR	Recurrent Middle-ear effusion *Streptococcus pneumoniae*	Biofilm formation inhibition Quantitative assay—microtiter plate assay CV staining and Spectrophotometry OD_540_	Exposure to xylitol lowered OD values, which were used as an indication of biofilm, compared with control BHI medium, but when the medium was supplemented with glucose or fructose, biofilm formation was enhanced and the inhibitory effect of xylitol on biofilm formation was not observed.
Yadav, 2015 South Korea [22]	Experimental study In vitro In vivo (otitis media-rat model)	Low	0.01%, 0.02%, 0.04%, and 0.08% Eugenol (Major component of clove oil) Carvacrol (Constituent of oregano)	n = 10 NR NR	Otitis media (not specified) Ear discharge MRSA and MSSA	Biofilm formation inhibition Biofilm eradication Rate of killed bacteria within biofilm Synergistic effect with carvacrol Quantitative assay—microtiter plate assay, CV staining, Spectrophotometry OD_570_, SEM, CFU count, In vivo colonization using OM rat model, FICI calculation	1. At Eugenol concentrations 0.01% and 0.02%, the decrease in biofilm growth was significant and dose-dependent, reaching complete inhibition of biofilm formation at 0.04% and 0.08% concentrations. Eugenol at MIC significantly decreased the biomass of already established biofilms by more than 50%. SEM showed that the cell aggregation and cell-to-cell connections were prevented, resulting in loosely arranged cells that can be easily disrupted. 2. A decrease of four log 10 steps in the number of viable cells was observed in biofilm treated with 2 × MIC eugenol. The MBEC of eugenol against biofilms from MRSA and MSSA clinical strains was found to be twice the MIC value. Eugenol kills or inhibits bacterial growth, consequently with low cells decreased biofilms. 3. In vivo model—sub-MIC of eugenol significantly decreased 88% *S. aureus* colonization in rat middle ear. The SEM images of the middle ears of rats showed the ciliated epithelium in the hypotympanum area and Eustachian tube orifice area are intact, here was no visible biofilms formation or detectable cell debris in the middle ear. 4. When eugenol and carvacrol were used in combination, the MBEC was reduced ≥ 4-fold, producing a synergistic effect on the eradication of pre-established biofilms as defined by FICI ≤ 0.5.
Rehman, 2016 Pakistan [23]	Experimental study In vitro	Low	Plant crude extracts (100 mg/mL) of Aloe barbadensis (aloe vera), Zingiber officinale (ginger), Curcuma longa (turmeric), and Acacia arabica (kikar).	n = 4 NR NR	Otitis media (not specified) Ear swab *Pseudomonas aeruginosa*, *Staphylococcus haemolyticus*, *Staphylococcus hominis*	Biofilm formation inhibition Qualitative assay-Congo red agar Quantitative assay-microtiter plate assay, Spectrophotometry OD_578_	Ethanolic and methanolic extract of *A. arabica* were found to be most effective in the inhibition of bacterial biofilm formation (concentrations from 1 to 15 mg/mL), particularly on *P. aeruginosa* biofilm.
Domenech, 2017 Spain [24]	Experimental study In vitro	Low	NAC, potent thiol-containing antioxidant, and Cys, an antioxidant	n = 1 Child NR	Acute otitis media NR NESP, NTHI	Biofilm eradication CLSM and specific fluorescent labeling of pneumococcal cells with Helix pomatia agglutinin	1. At a concentration of 0.5 mg/mL, in mixed biofilm, NAC killed 99% of NESP cells and eliminated NTHI. At 2.5 mg/mL (the MIC for planktonic cultures), bacteria were virtually eradicated. 2. Cys caused a 90% reduction in the viability of both pathogens when used at 0.5 and 2.5 mg/mL. A concentration of 5 mg/mL led to the almost total killing of NTHI and to the survival of just 2% of NESP cells.
Otsuka, 2017 USA [25]	Experimental study In vitro	Low	Antisense PNAs synthetic polymers, mimic DNA/RNA	n = 21 NR NR	Chronic otitis media Middle ear fluid and nasopharyngeal isolates NTHI	Biofilm eradication MBEC assay	MBECs were in a wide range, from 45 mg/l (10 µmol/L) up to 179 mg/L (40 µmol/L) for clinical isolates.
Bidossi, 2018 Italy [26]	Experimental study In vitro	Low	Probiotics *Streptococcus salivarius* 24SMB and *Streptococcus oralis* 89a (α-hemolytic streptococci isolated from the human pharynx of healthy individual)	NR NR NR	Upper respiratory tract infection (including otitis media) NR *S. aureus*, *Staphylococcus epidermidis*, *Streptococcus pyogenes*, *S. pneumoniae*, *Moraxella catarrhalis* *Propionibacterium acnes*	Biofilm formation inhibition Biofilm eradication Quantitative assay—microtiter plate assay, Spectrophotometric assay OD_595_ CLSM	1. The mixture of *S. salivarius* 24SMB and *S. oralis* 89a displayed an inhibitory activity against biofilm development of all tested bacteria, except for *S. pyogenes* whose biofilm formation was not significantly influenced by presence of the probiotic strains. 2. The combination of probiotics was able to significantly disrupt the pre-formed biofilm of all tested bacteria. Conversely, *S. pyogenes* biofilm was only slightly affected by the probiotic strains.
De la Torre Gonzalez, 2018 Mexico [27]	Case-control In vivo	Low	Commercially available 4% Sodium 2—MESNA	n = 10 cholesteatoma patients undergoing surgery Controls: cochlear implant surgery Cases: median 10 years (6–17) range 8/2 Controls: NR 2 females	Chronic otitis media with cholesteatoma NR NR	Biofilm eradication CLSM, LIVE/DEAD BacLight^®^ staining	Biofilm disaggregation: Complete biofilm disaggregation in 3 (30%) patients, partial disaggregation, and lower density of biofilm in 7 (70%) patients.
Jun, 2019 South Korea [28]	Experimental study In vitro	Low	NAC, potent thiol-containing antioxidant	n = 4 NR NR	Post-tympanostomy tube otorrhea Ear swab MRSA, QRPA	Bacterial adhesion, Biofilm formation inhibition, Biofilm eradication Quantitative assay—microtiter plate assay, SEM on tympanostomy tube	1. NAC significantly decreased the adhesion of MRSA and QRPA in a concentration-dependent manner (*p* < 0.01) and significantly inhibited the rate of biofilm formation by MRSA and QRPA strains at all concentrations (*p* < 0.001). The rate of biofilm formation by MRSA and QRPA strains. decreased equally at each concentration of NAC 2. NAC significantly increased the eradication rate of preformed MRSA and QRPA biofilms (*p* < 0.001). The rate of MRSA biofilm eradication increased as the exposure concentration of NAC increased. The rate of biofilm eradication from QRPA was seen equally at each concentration of NAC.
Gronseth, 2020 Norway [29]	Experimental study In vitro	Low	BAG S53P4 (Commercially available smaller granules of BAG-S53P4, <45 mm)	n = 10 NR NR	Otitis media (not specified, draining ear patients) Ear swab *S. aureus*	Biofilm eradication BOAT, BBT, CLSM with LIVE/DEAD staining	Exposure to 48 h primed BAG granules (100 mg/mL) produced bactericidal effects in all strains (*p* = 0.001), and CLSM showed a reduction of viable bacteria in biofilm (*p* = 0.001). Supernatant primed 14 days, 400 mg/mL, reduced metabolic activity (*p* < 0.001), showed bactericidal effects for all strains (*p* = 0.001), and CLSM showed fewer viable bacteria (*p* = 0.001). The supernatant primed for 48 h, or in concentrations lower than 400 mg/mL at 14 days, did not completely eradicate biofilm.
Abed, 2021, Iraq [30]	Experimental study In vitro	Low	Green synthesized CuO NPs	NR NR NR	Chronic otitis media Ear swab *S. aureus*, *P. aeruginosa*, *K. pneumoniae*, *S. epidermidis*, *E. coli*, *P. vulgaris*, *C. freundii*, *E. cloacae*, *H. influenzae*, *P. oryzihabitans*	Biofilm formation inhibition Qualitative assay-Congo red agar	1. Nanoparticle inhibited the production of biofilm 2. The effect was more obvious on Gram-negative bacteria.
Abed, 2021 Iraq [31]	Experimental study In vitro	Low	Radish (Raphanus sativus) root peel extracts (50% (*v*/*v*), winter food crop	NR NR NR	Otitis media (not specified) Ear swab *S. aureus*, *Citrobacter* spp., *Proteus* spp., *Klebsiella* spp., *Pseudomonas* spp., *Enterobacter* spp., *E. coli*, *Morganella* spp., *Aeromonas* spp.	Biofilm formation inhibition Qualitative assay-Congo red agar	Biofilm production was the factor most affected by the active substances of the extracts.
Mustafa, 2021 Egypt [32]	Experimental study In vitro	Low	AgNPs, made by direct sunlight irradiation on AGE	n = 5 NR NR	Otitis media (not specified) NR *Bacillus cereus*, *P. aeruginosa*, *Penicillium chrysogenum*, *Aspergillus niger*, *Aspergillus flavus*	Biofilm formation inhibition Qualitative assay-Congo red agar TEM	The obtained AgNPs showed highly significant (*p* value < 0.001) antibiofilm activities against tested strains. TEM images of *P. aeruginosa* and *A. flavus* treated with 25 μg/mL AgNPs showed shrinkage in the cytoplasmic materials and rupture of cell walls.
Bozic, 2023 Serbia [33]	Experimental study In vitro	Low	NAC/dry propolis extract fixed combination (200 mg NAC/80 mg dry propolis extract)	n = 29 mean 42.7 years (18–66) range NR	Chronic suppurative otitis media Middle ear mucosa biopsy *S. aureus*, *M. catarrhalis*, *S. pneumoniae*, *S. epidermidis*, *P. aeruginosa*, *H. influenzae*	Biofilm formation inhibition Biofilm eradication Quantitative assay—microtiter plate assay, Spectrophotometric assay OD_570_	1. Subinhibitory (1/2 to 1/8 MIC) concentrations reduced biofilm formation at all applied concentrations (*p* < 0.05). The effect was dose-dependent, a concentration of 1/2 MIC completely prevented biofilm formation in 80.0% of isolates. 2. Suprainhibitory concentrations reduced preformed biofilm at all concentrations applied (*p* < 0.05), and the effect was dose-dependent. The most effective concentration was 8 × MIC, which resulted in complete biofilm eradication in 91.4% of isolates and category reduction in an additional 5.7% of isolates (*p* < 0.05).
Artono, 2023 Indonesia [34]	Experimental study In vitro	Low	Acetic acid (concentration 0.04%, 0.08%, 0.16%, 0.31%, 0.63%, 1.25%, 2.5%, 5%).	n = 5 NR NR	Chronic suppurative otitis media Ear swab *P. aeruginosa*	Biofilm formation inhibition Biofilm eradication Quantitative assay—microtiter plate assay, Spectrophotometric assay	1. The biofilm inhibitory effect of acetic acid on *P. aeruginosa* was obtained with significant results at concentrations of 0.16%, 0.31%, 0.63%, 1.25%, 2.5%, and 5%. The MBIC value was 0.16%. 2. The effect of acetic acid on *P. aeruginosa* biofilm eradication was obtained with significant results (*p* < 0.05) at concentration groups of 0.08%, 0.16%, 0.31%, 0.63%, 1.25%, 2.5%, and 5%. The MEBC was 0.08%.

NR, not reported; MRSA, methicillin-resistant *Staphylococcus aureus*; SEM, scanning electron microscopy; CV, crystal violet; OD, optical density; BHI, brain heart infusion; MSSA, methicillin-susceptible *Staphylococcus aureus*; CFU, colony forming unit; MIC, minimum inhibitory concentration; OM, otitis media; FICI, fractional inhibitory concentration index; MBEC, minimum biofilm eradication concentration; NAC, N-acetyl L-cysteine; Cys, cysteamine; NESP, non-encapsulated *Streptococcus pneumoniae*; NTHI, non-typeable *Haemophilus influenzae*; CLSM, confocal laser scanning microscopy; PNA, peptide nucleic acids; DNA, deoxyribonucleic acid; RNA, ribonucleic acid; MESNA, mercaptoethanesulfonate; QRPA, quinolone-resistant *Pseudomonas aeruginosa*; BAG, bioactive glass; BOAT, biofilm-oriented antiseptic test; BBT, biofilm bactericidal test; CuNPs, copper oxide nanoparticles; AgNPs, silver nanoparticles; AGE, aqueous garlic extract; TEM, transmission electron microscopy; MBIC, minimum biofilm inhibitory concentration.

**Table 3:** Risk of bias assessment table (JBI critical appraisal tool for quasi-experimental studies).

Author, Year	Q1	Q2	Q3	Q4	Q5	Q6	Q7	Q8	Q9	Total	Risk of Bias %
Park, 2007 [20]	√	x	x	x	√	√	√	√	x	5/9	55.6
Kurola, 2010 [21]	√	x	x	x	√	√	√	√	√	6/9	66.7
Yadav, 2015 [22]	√	√	x	x	√	√	√	√	√	7/9	77.8
Rehman, 2016 [23]	√	√	√	x	√	√	√	√	√	8/9	88.9
Domenech, 2017 [24]	√	x	x	x	√	√	√	√	x	5/9	55.6
Otsuka, 2017 [25]	√	x	√	x	x	√	√	√	x	5/9	55.6
Bidossi, 2018 [26]	√	x	√	x	x	√	√	x	√	5/9	55.6
De la Torre Gonzalez, 2018 [27]	√	x	x	x	√	√	√	x	√	5/9	55.6
Jun, 2019 [28]	√	x	√	x	√	√	√	√	√	7/9	77.8
Gronseth, 2020 [29]	√	x	√	x	√	√	√	√	√	7/9	77.8
Abed, 2021 [30]	√	x	x	x	?	√	√	√	√	5/9	55.6
Abed, 2021a [31]	√	x	x	x	√	√	√	√	√	6/9	66.7
Mustafa, 2021 [32]	√	x	x	x	N/A	√	√	√	√	5/9	55.6
Bozic, 2023 [33]	√	x	x	x	√	√	√	√	√	6/9	66.7
Artono, 2023 [34]	√	x	√	x	√	√	√	√	√	7/9	77.8

Abbreviations: √—yes, x—no, ?—unclear, N/A—not applicable, JBI—the Joanna Briggs Institute.

**Table 4:** Feasibility assessment.

Component	Evaluation and Justification
Research Question and Objectives	The research question was defined as follows: Does any novel natural or synthetic compound have a more frequent antibiofilm effect on bacterial biofilm formed from clinical isolates taken from patients with otitis media in comparison with standard therapeutic antimicrobial agents or untreated biofilms in these cases? Objectives: Primary—To evaluate the difference in frequency of antibiofilm effect of the novel compound on bacterial biofilm formed from clinical isolates originating from patients with otitis media in comparison with the standard therapeutic approach or placebo. Secondary—To perform primary aim within subgroups of otitis media types and causative agents, depending on previous antibiotic treatment, genders, and age groups.
Availability of Data	Search Three electronic databases were searched (PubMed, SCOPUS, and Web of Science), and a total of 1747 articles were screened in the first step (title and abstract reading). Inclusion criteria: Population—all human patients with bacterial otitis media Intervention—novel natural or synthetic compound with an antibiofilm effect Control—standard therapeutic antimicrobial agents or untreated biofilms Outcome—antibiofilm effect (inhibition, eradication) Study design—experimental study, controlled trials, prospective or retrospective cohort design, nested case-control in cohort design, case-control design, and cross-sectional design Exclusion criteria: Foreign language—articles published in other language than English Wrong publication type—if the publication was not an original article Wrong population—cases were not humans with otitis media Wrong method—no biofilm detection Wrong outcome—no antibiofilm effect evaluated Wrong topic—none of the inclusion criteria met 15 articles (14 in vitro and 1 in vivo with control group) were included in qualitative synthesis.
Homogeneity of Studies	There was high heterogeneity of included studies regarding: compounds (“A total of 17 different novel compounds were examined in 15 included studies.”) study population (“Commonly evaluated type of OM was chronic otitis in six studies of which two examined chronic suppurative form of otitis. Recurrent and acute otitis were examined in one study each. The type of otitis was not specified in seven included publications.”) causative agents (“The antibiofilm effect (biofilm inhibition and/or biofilm eradication) of novel compounds were evaluated on 28 different causative agents that were isolated from clinical samples.”) outcomes (“Biofilm inhibition only by an examined compound was assessed in six studies; biofilm eradication only was examined in four studies, while both biofilm inhibition and biofilm eradication were examined in five studies. It was established that examined compound had a dose-dependent effect in seven studies.”)
Methodological Quality	Was evaluated by the New Castle Ottawa scale system and JBI risk of bias tool for quasi-experimental studies. All 15 included studies had low quality. Moderate risk of bias was present in 10, and low in 5 studies
Heterogeneity Assessment	Major findings were so heterogeneous that the overall conclusion cannot be drawn.
Statistical Feasibility	There was no OR, RR, HR or other effect measured due to the absence of a control group.
Publication Bias	Because of the high heterogeneity between included studies, the decision has been made to perform just a systematic review.
Resource Availability	Technical resources (three electronic databases), human resources (two independent reviewers with the third for solving disagreements)
Ethical Considerations	There were no ethical conflicts within the included studies.
Practicality of Results	The results of the meta-analysis will imply the purpose and effectiveness of novel compounds; however, in situations of high heterogeneity within studies and scarcity of data derived from the control group, a systematic review will obtain significant results for future research.

## Data Availability

Not applicable.

## Supplementary Materials

The following supporting information can be downloaded at: https://www.mdpi.com/article/10.3390/ijms252312841/s1.
